# Reduced Coupling Between Blood‐Oxygen‐Level‐Dependent Signal and Cerebrospinal Fluid Flow in Obsessive‐Compulsive Disorder

**DOI:** 10.1002/cns.71232

**Published:** 2026-11-09

**Authors:** Jinqi Liu, Ziyun Xu, Wanting Liu, Carol A. Seger, Ziwen Peng, Gangqiang Hou

**Affiliations:** ^1^ Center for the Study of Applied Psychology, Guangdong Key Laboratory of Mental Health and Cognitive Science and School of Psychology South China Normal University Guangzhou China; ^2^ Key Laboratory of Brain, Cognition and Education Sciences, Philosophy and Social Science Laboratory of Reading and Development in Children and Adolescents (South China Normal University) Ministry of Education Guangzhou China; ^3^ Neuropsychiatry Imaging Center, Department of Radiology, Shenzhen Mental Health Center Shenzhen Kangning Hospital Shenzhen Guangdong China; ^4^ Department of Psychology Colorado State University Fort Collins Colorado USA

**Keywords:** BOLD–CSF coupling, glymphatic system, obsessive‐compulsive disorder

## Abstract

**Aims:**

Mounting evidence implicates inflammation in the pathogenesis of obsessive‐compulsive disorder (OCD). Given the interaction between inflammation and the glymphatic system, we aimed to investigate whether altered glymphatic function is present in patients with OCD.

**Methods:**

We assessed glymphatic‐related CSF dynamics using resting‐state functional MRI (rs‐fMRI) in 53 patients with OCD and 74 healthy controls. We measured global coupling between cortical blood‐oxygen‐level‐dependent signal and ventricular cerebrospinal fluid flow, along with the time lags. Given that the goal‐directed and habitual learning framework has become a dominant neurobiological model for understanding the development and maintenance of obsessive‐compulsive symptoms, we also examined regional couplings and lags within the goal‐directed and habitual learning networks.

**Results:**

Patients with OCD exhibited weaker BOLD–CSF coupling and negative derivative BOLD–CSF coupling strength than HC, both globally and regionally within the goal‐directed and habitual systems. OCD patients also showed a longer time lag of global BOLD–CSF coupling.

**Conclusion:**

These findings suggest that altered glymphatic‐associated CSF dynamics are present in OCD, offering critical new insights into the underlying mechanisms of the disorder.

## Graphical abstract

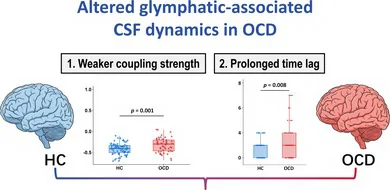

## 1 Introduction

Obsessive‐compulsive disorder (OCD) is a common psychiatric disorder affecting 2%–3% of the population and associated with substantial disability [1]. Characterized by excessive obsessions and compulsions, OCD is also associated with reduced subjective well‐being [2]. Emerging evidence indicates that inflammation [3, 4, 5] and chronic oxidative stress [6, 7] contribute to OCD pathogenesis. Inflammation‐related OCD is exemplified by pediatric acute‐onset neuropsychiatric syndrome (PANS) and childhood acute neuropsychiatric syndrome (CANS), while studies of primary OCD further support the involvement of neuroimmune mechanisms [8]. Increased plasma levels of IL‐2, IL‐4, IL‐6, IL‐10, and TNF‐α have been reported in medication‐naïve OCD patients without psychiatric comorbidities [9], and early‐onset OCD is associated with altered peripheral monocyte profiles and increased proinflammatory cytokine production [4].

The glymphatic system is a brain‐wide pathway that facilitates cerebrospinal fluid (CSF) transport and metabolic waste clearance [10, 11], and its dysfunction is closely linked to inflammatory and immune abnormalities. CSF flows through perivascular spaces and exchanges with interstitial fluid via astrocytic aquaporin‐4 (AQP‐4) water channels [12], facilitating the clearance of neurotoxic metabolites. Glymphatic impairment promotes toxic protein accumulation and neuroinflammation, which further disrupts fluid exchange and establishes a vicious cycle of impaired clearance and inflammation [13, 14]. Altered glymphatic function has been widely observed in neurodegenerative disorders, including Alzheimer's and Parkinson's diseases [15, 16]. Increasing evidence also implicates glymphatic dysfunction in psychiatric disorders, including major depressive disorder [17], schizophrenia [18], and bipolar disorder [19], and links glymphatic alterations to cognitive dysfunction [20, 21]. Moreover, impaired glymphatic function is associated with psychiatric vulnerability factors, including acute stress responses, chronic inflammation, and sleep disturbances [6, 22, 23].

Recent research has proposed a mechanistic pathway whereby peripheral infection induces central neuroinflammation and subsequent glymphatic impairment in psychiatric disorders [24, 25]. Although direct investigations of glymphatic function in OCD have not been published, preliminary evidence indicates elevated CSF total protein levels [26], increased glutamate levels [27], and enlarged basal ganglia perivascular spaces in late‐onset OCD patients [28]. These findings suggest that disrupted interactions between glymphatic function and neuroinflammation may be present in patients with OCD.

Several MRI‐based approaches have been developed to assess glymphatic function, including diffusion tensor imaging analysis along the perivascular space (DTI‐ALPS) and enlarged perivascular space (ePVS) quantification. Among these methods, resting‐state fMRI‐derived blood‐oxygen‐level‐dependent signal and ventricular cerebrospinal fluid coupling (BOLD–CSF coupling) provides an indirect measure of CSF dynamics driven by hemodynamic oscillations [29]. Cerebrospinal fluid (CSF), as the primary carrier of glymphatic clearance, exhibits large inflow waves approximately every 20 s during sleep [30]. Based on the principle of constant intracranial volume [31], as BOLD signal decreases, CSF volume should increase, resulting in a negative correlation between BOLD and CSF inflow signals. This coupling reflects CSF inflow efficiency driven by hemodynamic oscillations. In addition, system responsiveness is characterized by two features of BOLD–CSF coupling: strength and temporal delay (time lag). Strength is quantified by the BOLD–CSF correlation and the negative‐derivative BOLD–CSF correlation. Because the negative derivative isolates the descending phase of the BOLD signal—when cerebral blood volume decreases and, under constant intracranial volume, drives compensatory CSF influx—this metric specifically reflects glymphatic CSF inflow efficiency driven by hemodynamic oscillations. Time lag is defined as the time point corresponding to the extreme correlation value (i.e., for the BOLD–CSF correlation or the negative‐derivative BOLD–CSF correlation). The associated time lags of these correlations reflect the time required for vascular oscillations to propagate to ventricular CSF flow [30, 32].

The metrics of BOLD–CSF coupling strength and the corresponding time lag are considered to reflect glymphatic function, the impairment of which has been associated with AD pathology as well as cognitive impairment in Parkinson's disease [33]. In addition, spatial analyses of BOLD–CSF coupling strength have demonstrated regional heterogeneity, with stronger negative coupling observed in regions with high cerebral blood perfusion such as the visual cortex [32]. Recent studies have therefore examined regional BOLD–CSF coupling to characterize glymphatic‐related dynamics across distinct brain regions [21]. This regional specificity motivated our selection of BOLD–CSF coupling as the primary imaging marker in the present study and decision to examine these measures within goal‐directed and habitual learning systems. Neuroimaging studies have consistently demonstrated structural and functional abnormalities within the goal‐directed and habitual learning systems in OCD [34]. The habitual learning system is primarily characterized by alterations in the putamen and supplementary motor area, whereas the goal‐directed system centers on the caudate and medial prefrontal cortex [35]. The goal‐directed system supports flexible action selection based on expected outcomes, whereas the habitual system mediates automatic stimulus—response behaviors that are relatively insensitive to outcome value [36]. Dysfunction within these systems has been associated with symptom severity, illness duration, and age of onset. In OCD, impaired goal‐directed control is accompanied by excessive reliance on habitual mechanisms [37]. Gillan and Robbins proposed that OCD symptoms may emerge through a progression from early goal‐directed deficits to increasing habitual dominance, contributing to compulsive behavior persistence [35]. The goal‐directed–habitual system model provides a framework for understanding the development and maintenance of obsession and compulsivity by emphasizing the imbalance between flexible control and automatic responses. Moreover, because regional hemodynamic oscillations may be influenced by local neural fluctuations, integrating multiple regions (e.g., caudate, dlPFC, vmPFC, dACC, and vlPFC for the goal‐directed system; putamen, SMA, and insula for the habit‐learning system) into dual‐system‐level ROIs may provide more robust coupling estimates [38, 39, 40, 41]. Therefore, we investigated whether regional BOLD–CSF coupling, as a marker of glymphatic‐related function, is altered within the goal‐directed and habitual learning systems in OCD.

The present study assessed both global and regional (goal‐directed and habitual systems) BOLD–CSF coupling in patients with OCD and healthy controls. We hypothesized that patients with OCD would exhibit weaker BOLD–CSF coupling and weaker negative derivative BOLD–CSF coupling strengths than HCs, both globally and regionally within the goal‐directed and habitual systems. Additionally, we hypothesized that patients with OCD would show a longer temporal lag compared with healthy controls, both globally and regionally.

## 2 Methods

### 2.1 Participants

We recruited 63 patients with obsessive‐compulsive disorder (OCD) and 82 healthy controls from Kangning Hospital, Shenzhen, Guangdong Province. Patients were eligible if they were aged 18–55 years and met DSM‐5 criteria for OCD, confirmed by a research psychiatrist, with comorbidities assessed using the Mini International Neuropsychiatric Interview (MINI). Exclusion criteria included MRI contraindications, a history of brain injury, severe cognitive impairment, severe somatic disease, substance abuse, and pregnancy or lactation. Comorbid psychiatric disorders were permitted when OCD was the primary diagnosis and included depression (*n* = 3), anxiety disorders (*n* = 2), schizophrenia and anxiety disorders (*n* = 1), schizophrenia (*n* = 1), and body dysmorphic disorder (*n* = 1). Healthy controls were aged 18–55 years, had no DSM‐5 psychiatric diagnosis, and provided written informed consent. They were additionally excluded if MINI screening indicated a family history of psychiatric disorders or current use of psychotropic medications.

Six patients with OCD and 10 healthy controls were excluded due to excessive head motion, and two additional healthy controls were excluded due to incomplete BOLD signal time series. The final sample included 53 patients with OCD and 74 healthy controls. Patients with OCD had a mean age of onset of 19.81 years and a mean illness duration of 8.11 years. Twenty‐four patients were receiving stable pharmacotherapy, predominantly SSRIs (e.g., sertraline, fluvoxamine, and paroxetine), either alone or combined with atypical antipsychotics (e.g., olanzapine, quetiapine, and clozapine), benzodiazepines, or mood stabilizers. Eighteen patients received multiple psychotropic medications. Sensitivity analyses addressing the potential effects of medication use, age of onset, and illness duration are provided in Table 1 and Figures S1 and S2.

**TABLE 1:** ROI classification for goal‐oriented and habit‐based learning systems.

Goal‐directed		Habit‐learning	
ROI	AAL3 labels	ROI	AAL3 labels
Caudate L, R	75, 76	insula L, R	33, 34
dACC L, R	37, 38	PoCG L, R	61, 62
dlPFC L, R	3, 4, 5, 6	PreCG L	1, 2
dmPFC	19, 20	putamen L, R	77, 78
IFC L, R	9, 10	SMA L, R	15, 16
IPC L, R	65, 66		
OFC L, R	27, 28, 29, 30, 31, 32		
supACC L, R	155,156		
vlPFC L, R	7,8,11,12		
vmPFC L, R	21,22,23,24,25,26		

*Note:* The parcellation was performed on the 1 mm resolution AAL3v1 template (https://www.oxcns.org/aal3.html).

Abbreviations: L, left; R, right.

### 2.2 Clinical Assessments

Clinical measures included the Obsessive‐Compulsive Inventory–Revised (OCI‐R) [42]; the Beck Depression Inventory (BDI) [43] to assess the severity of depressive symptoms; and the State–Trait Anxiety Inventory (STAI) [44]. The Yale‐Brown Obsessive‐Compulsive Scale (Y‐BOCS) [45] was assessed by trained clinicians to evaluate the severity of OCD symptoms. Y‐BOCS scores ≥ 16 were considered indicative of moderate‐to‐severe symptom severity, consistent with severity classifications proposed by the Stanford OCD Program and other literature [46].

### 2.3 Resting‐State fMRI Data Acquisition and Preprocessing

#### 2.3.1 Acquisition of Resting‐State fMRI Data

All participants underwent resting‐state functional MRI at Kangning Hospital, Shenzhen. During scanning, participants were instructed to keep their eyes closed and remain awake. Resting‐state fMRI data were acquired using a 3.0‐T Discovery MR750 scanner (GE Healthcare, USA) equipped with an eight‐channel phased‐array head coil and a gradient‐echo EPI sequence. The acquisition parameters were as follows: repetition time/echo time (TR/TE) = 2000/25 ms; flip angle = 90°; slice thickness = 3.2 mm with no inter‐slice gap; acquisition matrix = 64 × 64; field of view (FOV) = 220 × 220 mm^2^; 48 axial slices; and 240 volumes (8 min). High‐resolution structural images were acquired using a three‐dimensional T1‐weighted BRAVO sequence with the following parameters: inversion time (TI) = 450 ms; flip angle = 12°; acquisition matrix = 256 × 256; TR = 6.7 ms; TE = 2.9 ms; FOV = 256 × 256 mm^2^; and 196 sagittal slices covering the whole brain without inter‐slice gaps (slice thickness = 1.0 mm).

#### 2.3.2 Preprocessing of Resting‐State fMRI Data

Resting‐state fMRI data were preprocessed using Data Processing & Analysis for Brain Imaging (DPABI, http://rfmri.org/DPARSF), which is based on Statistical Parametric.

Mapping (SPM12, http://www.fil.ion.ucl.ac.uk/spm), implemented in MATLAB R2024b.

Preprocessing in this study largely followed procedures used in prior studies [47, 48], and included: (i) removal of the first 10 volumes for signal stabilization; (ii) slice timing correction; (iii) realignment for head motion correction; (iv) skull stripping; (v) linear regression of white matter signals; (vi) linear detrending; and (vii) temporal band‐pass filtering (0.01–0.1 Hz). For CSF signal preprocessing, motion correction was not applied because it would disrupt voxel‐layer positional information required for inflow analysis [30].

Following previous BOLD–CSF coupling studies, global BOLD (gBOLD), CSF signals, and motion parameters were not regressed out, as coupling estimation requires preservation of gBOLD–CSF relationships and motion regression may attenuate global BOLD signals [30]. Spatial smoothing was not performed to preserve spatial resolution. In sensitivity analyses, head motion measured as mean framewise displacement (FD) was included as a nuisance covariate in all group comparisons and correlation analyses. Participants with excessive head motion (rotation > 2.5° or translation > 2.5 mm) were excluded (6 patients with OCD and 10 healthy controls).

### 2.4 Definition of Masks

Cortical gray‐matter ROIs were defined using the Harvard–Oxford cortical and subcortical structural atlases (https://neurovault.org/collections/262/). ROIs representing the goal‐directed and habitual learning systems were defined based on 1‐mm resolution AAL3 atlas coordinates from previous work [49]. Given the limited evidence linking the middle temporal gyrus (MTG) to the habit‐learning system, we excluded the MTG from the habit‐learning ROIs derived from that study, with corresponding labels provided in Table 1. T1‐weighted images were coregistered to rs‐fMRI images and jointly normalized to MNI space using SPM12 to generate deformation fields. MNI‐space ROIs were then transformed into native rs‐fMRI space to generate masks for extracting BOLD signals. The CSF ROIs were manually defined in native space on the lowermost rs‐fMRI slices adjacent to the cerebellar base to encompass the 4th ventricle and capture CSF inflow effects [31], with anatomical confirmation using coregistered T1‐weighted images (Figure 1). ROI definition procedures followed previous studies [16].

**FIGURE 1:**
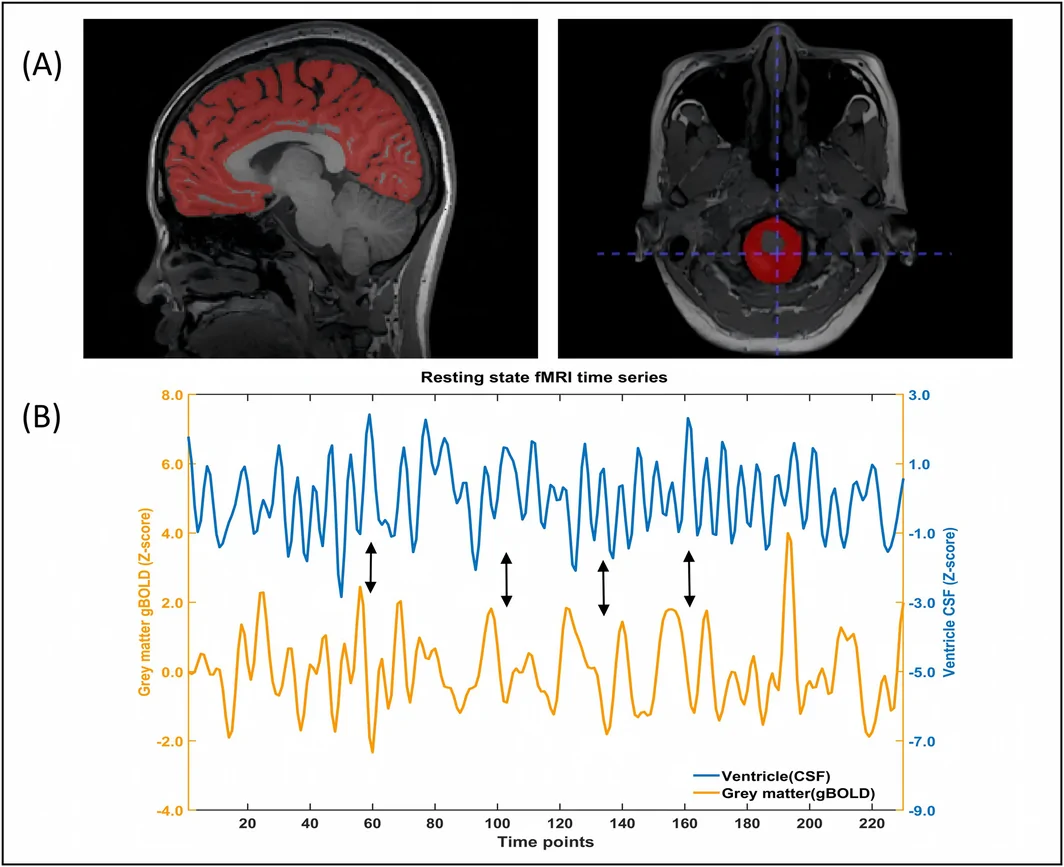
Schematic of the masks (single subject). (A) Left: Mask used to extract gBOLD signals; Right: Mask used to extract CSF signals. (B) Example time series of cortical gBOLD signal (red line) and CSF flow signal (blue line) from one participant. Note that the two signals showed corresponding amplitude fluctuations (black arrows).

### 2.5 Extraction of CSF Inflow Signal and rs‐fMRI Signal

The global BOLD (gBOLD) signal was extracted from the transformed Harvard‐Oxford mask. Before voxel‐wise averaging, signals from individual voxels were z‐scored to ensure equal contribution across voxels. The resulting gBOLD amplitude reflects whole‐brain synchronization. Two healthy controls were excluded due to failed gBOLD signal extraction. Signals from the goal‐directed and habitual learning systems masks were extracted using the same procedure.

The CSF signal was extracted from manually delineated CSF masks, averaged across voxels, and normalized to characterize overall CSF inflow intensity and temporal dynamics. To assess the reliability of the CSF ROI delineation, masks were independently drawn for each participant by two research team members who independently completed the manual delineations. The intraclass correlation coefficient (ICC) of BOLD–CSF coupling strength between the two delineations was 0.988 (*p* < 0.001), indicating consistency between the two definitions of the cerebrospinal fluid ROI.

### 2.6 Quantifying Global and Regional BOLD–CSF Coupling

We computed gray matter–cerebrospinal fluid (CSF) signal coupling strength separately for the whole brain, goal‐directed system, and habitual learning system. Using MATLAB R2024b, Pearson cross‐correlations were calculated between the BOLD signals from the whole brain and each system and the CSF signal across lag times from −10 to +10 s [16]. In addition, negative‐derivative BOLD–CSF coupling was calculated by first taking the first‐order temporal derivative of the BOLD time series, multiplying it by −1, and setting negative values to zero to retain the descending phases of BOLD fluctuations, following previous approaches [30, 47]. The processed derivative signal was then cross‐correlated with the CSF signal across the same range of lag times.

As CSF volume increases, the BOLD signal is expected to decrease, resulting in an inverse relationship between the two measures. The original BOLD–CSF coupling measure captures the overall inverse relationship between BOLD and CSF signals, whereas the negative‐derivative measure specifically emphasizes CSF fluctuations associated with the descending phase of BOLD fluctuations. Accordingly, stronger conventional BOLD–CSF coupling is indicated by more negative correlation values, whereas larger negative‐derivative BOLD–CSF correlation values indicate stronger coupling. The corresponding formulas are:

(1) $$r\left(\tau \right)=\text{corr}\left(\text{BOLD}\left(t\right),\mathrm{CSF}\left(t+\tau \right)\right)$$

(2) $$D\left(t\right)=-\frac{\text{dBOLD}\left(t\right)}{dt}$$

(3) $$D^{\prime }\left(t\right)\left\{\begin{array}{cc} D\left(t\right), & D\left(t\right)>0\\ 0, & D\left(t\right)\leq 0 \end{array}\right.$$

(4) $$r_{D}\left(\tau \right)=\text{corr}\left(D^{\prime }\left(t\right),\mathrm{CSF}\left(t,\tau \right)\right)$$

where BOLD(*t*) denotes the BOLD signal at time *t*, CSF(*t*) denotes the CSF signal at time *t*, *τ* denotes the time lag, *r*(*τ*) denotes the Pearson correlation coefficient between BOLD and CSF signals at lag *τ*, and *r*_D_(*τ*) denotes the corresponding coefficient for the negative temporal derivative of the BOLD signal. For each participant, the lag *τ* corresponding to the minimum conventional BOLD–CSF correlation and the maximum negative‐derivative BOLD–CSF correlation was identified and used for subsequent between‐group comparisons. *D*′(*t*) represents the magnitude of the descending phase of BOLD fluctuations after inversion of the temporal derivative and removal of negative values.

BOLD–CSF coupling was assessed using 5000 permutations, with the CSF time series circularly shifted relative to the BOLD time series to generate null distributions.

### 2.7 Statistical Analysis

Group differences in demographic and clinical variables were assessed using independent‐samples *t*‐test, Mann–Whitney *U* tests, or *χ* ^*2*^ tests as appropriate. Spearman correlations were used to examine associations between BOLD–CSF coupling and age, head motion, and years of education. Multiple linear regression (MLR) models were used to examine group differences in BOLD–CSF coupling strength while controlling for age, sex, head motion, and years of education. Associations between demographic characteristics and coupling strength were examined after controlling for age, sex, education years, and head motion, with FDR correction applied to the correlation analyses. Intraclass correlation coefficients (ICC) were used to assess the reproducibility of CSF ROI delineation. Statistical significance was set at *p* < 0.05.

## 3 Results

### 3.1 Demographic and Clinical Characteristics

After excluding participants with excessive head motion (2.5 mm in displacement or 2.5° in rotation) or failed signal extraction, data from 74 healthy controls (HCs) and 53 patients with OCD were included in the final analysis. There were no significant between‐group differences in demographic variables, including sex, age, and years of education, between the HCs and OCD patients (*p* = 0.072; *p* = 0.091; *p* = 0.178). Descriptive statistics for other clinical scale scores are presented in Table 2. Scores on the Checking, Cleaning, Obsession, Ordering, and Control subscales of the OCI‐R are shown in Table S2 and showed significant between‐group differences, with OCD patients scoring significantly higher than HCs. No significant between‐group difference was found for the Hoarding subscale score.

**TABLE 2:** Comparison of basic demographic data and clinical characteristics.

	HC (*n* = 74)	OCD (*n* = 53)	*p*
Gender (M/F)	43/31	39/14	0.072
Age	27.22 ± 8.53	29.36 ± 8.43	0.091
Education years	13.92 ± 2.37	13.02 ± 3.25	0.178
Y‐BOCS total	5.32 ± 5.62	20.22 ± 7.76	< 0.001
Y‐BOCS obsession	2.97 ± 3.49	9.24 ± 5.35	< 0.001
Y‐BOCS compulsion	2.32 ± 3.03	7.60 ± 5.28	< 0.001
OCI‐R	12.08 ± 8.11	23.64 ± 12.75	< 0.001
OBQ	86.33 ± 30.54	117.19 ± 44.75	< 0.001
BDI	6.32 ± 6.29	16.04 ± 11.02	< 0.001
S_AI	36.39 ± 9.47	48.35 ± 12.89	< 0.001
T_AI	38.44 ± 9.34	52.08 ± 12.63	< 0.001

*Note: p*‐value after false discovery rate correction. Data are presented as mean ± standard deviation (M ± SD).

Abbreviations: BDI, Beck Depression Inventory; HC, healthy control; OBQ, obsessive beliefs questionnaire; OCD, obsessive‐compulsive disorder; OCI‐R, obsessive‐compulsive inventory revised; S_AI, state anxiety index; T_AI, trait anxiety index; Y‐BOCS, Yale‐Brown Obsessive‐Compulsive Scale (including obsession and compulsion subscores).

### 3.2 Between‐Group Comparison of Global BOLD–CSF Coupling Strength

In the average cross‐correlation curve across all participants from the combined HC and OCD groups (Figure 2, top left panel), the strongest negative correlation between the global BOLD (gBOLD) signal and the CSF signal occurred at *a* + 2 s time lag (*r* = −0.38, *t* = −27.334, *p* < 0.001; permutation test with *n* = 5000; Cohen's *d* = −2.43). The strongest significant positive correlation was observed at *a* − 8 s time lag (*r* = 0.33, *p* < 0.001). For the negative derivative of the gBOLD signal (Figure 2, top right panel), the strongest positive correlation with the CSF signal occurred at *a* − 4 s time lag (*r* = 0.47, *t* = 29.215, *p* < 0.001; permutation test with *n* = 5000; Cohen's *d* = 2.59). These correlations survived Bonferroni correction. Therefore, the +2 s time point was selected to extract individual global BOLD–CSF coupling strength, and the −4 s time point was selected to extract individual negative‐derivative gBOLD–CSF coupling strength for subsequent analyses. The same procedure was applied to the regional analyses, with the corresponding time points likewise selected for extracting regional coupling strengths (for a detailed explanation of the time points used to extract coupling strength, see Table S3).

**FIGURE 2:**
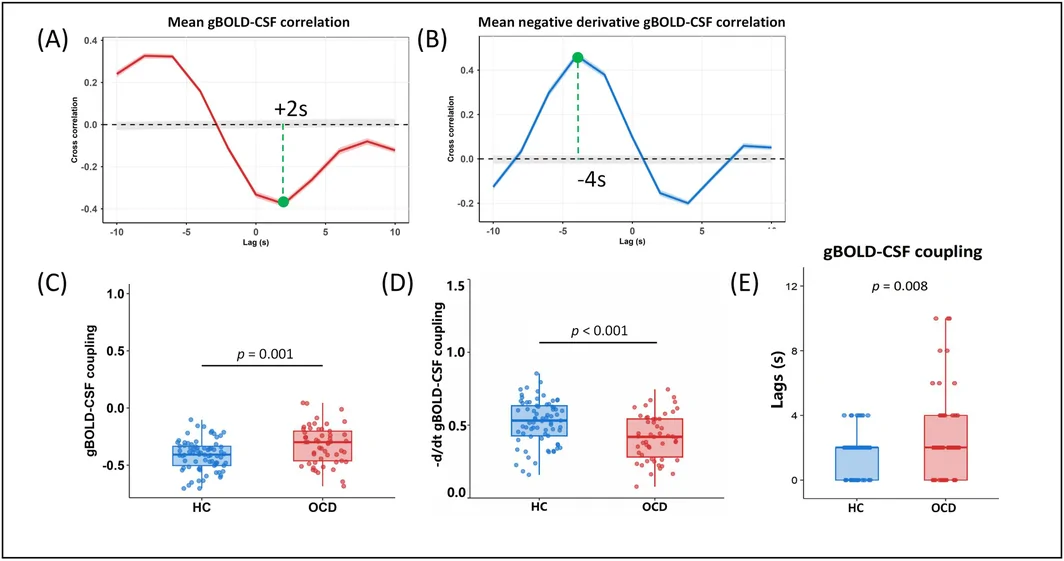
Between‐group comparison of global BOLD–CSF coupling. (A) Mean gBOLD–CSF cross‐correlation across all participants in the combined HC and OCD groups (red line). The maximum occurred at a time lag of −8 s, whereas the minimum occurred at +2 s. This measure, derived from the original signals, reflects the overall strength of BOLD–CSF coupling associated with CSF influx. The 95% permutation‐based null interval at a time lag of +2 s ranged from −0.016 to 0.023. The colored shaded band represents the mean ± standard error of the mean (SEM) across participants. (B) Mean negative‐derivative gBOLD–CSF cross‐correlation across all participants in the combined HC and OCD groups (blue line). The maximum occurred at a time lag of −4 s, whereas the minimum occurred at +4 s. This measure, based on the negative first‐order derivative of the BOLD signal, reflects BOLD–CSF coupling specifically during the declining phase of the BOLD signal. The 95% permutation‐based null interval at a time lag of −4 s ranged from −0.021 to 0.016. The colored shaded band represents the mean ± SEM across participants. (C) Between‐group comparison of gBOLD–CSF coupling strength. For negative correlations, larger absolute values indicate stronger coupling. (D) Between‐group comparison of negative derivative gBOLD–CSF coupling strength. For positive correlation values, larger values indicate stronger coupling. Boxes represent the interquartile range (25th–75th percentiles), with the horizontal lines indicating the median. Whiskers extend to 1.5 × the interquartile range. Individual dots represent individual subject values. (E) Between‐group comparison of the time lag for gBOLD–CSF coupling.

An independent‐samples *t*‐test revealed that the OCD group showed significantly weaker gBOLD–CSF coupling strength (Figure 2, bottom panel) than the HC group (Welch's *t* = −3.34, *p* = 0.001). A sensitivity analysis using participant‐specific time lags to derive negative‐derivative gBOLD–CSF coupling strength yielded a similarly significant between‐group difference (see Figure S3). The OCD group also showed significantly weaker negative derivative gBOLD–CSF coupling strength compared to the HCs (Welch's *t* = −3.11, *p* = 0.002). After controlling for age, sex, years of education, and head motion, both gBOLD–CSF coupling strength and negative‐derivative gBOLD–CSF coupling remained significantly lower in patients with OCD than in HCs (*t* = 2.60, *p* = 0.010; *t* = −3.20, *p* = 0.002, respectively). Patients with OCD exhibited a significantly longer time lag of gBOLD–CSF coupling than HCs (*t* = 2.73, *p* = 0.008) (Figure 2, bottom‐right panel). The time lag of negative‐derivative gBOLD–CSF coupling was not significantly longer in patients with OCD than in HCs (*t* = 0.84, *p* = 0.401).

### 3.3 Between‐Group Comparison of Regional BOLD–CSF Coupling Strength in Goal‐Directed and Habitual Learning Systems

Within the goal‐directed system (Figure 3A), significant between‐group differences were observed for regional BOLD–CSF coupling strength (*t* = −3.17, *p* = 0.002) and negative‐derivative regional BOLD–CSF coupling strength (*t* = 3.00, *p* = 0.003). Patients with OCD showed significantly weaker coupling than HCs on both measures. We found no significant group differences in the time lags of BOLD–CSF coupling and negative derivative BOLD–CSF coupling.

**FIGURE 3:**
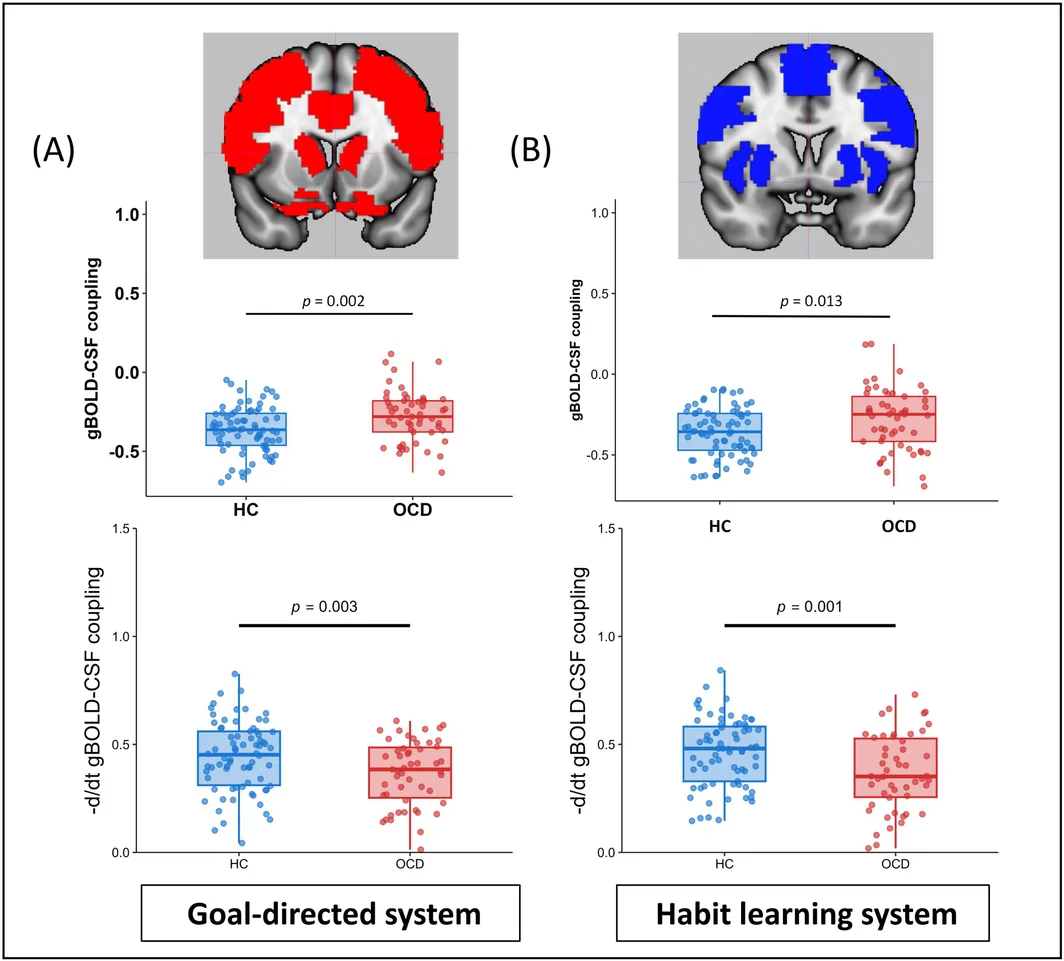
Between‐group comparison of regional BOLD–CSF coupling strength in the goal‐directed and habit learning systems. (A) Top panel: Red regions indicate brain areas within the goal‐directed system, shown in a coronal slice at *y* = 13 mm. Middle panel: Intergroup comparison of regional BOLD–CSF coupling strength in the goal‐directed system. Bottom panel: Intergroup comparison of regional negative derivative BOLD–CSF coupling strength in the goal‐directed system. (B) Top panel: Blue regions indicate brain areas within the habit‐learning system, shown in a coronal slice at *y* = 3 mm. Middle panel: Intergroup comparison of regional BOLD–CSF coupling strength in the habit learning system. Bottom panel: Intergroup comparison of regional negative derivative BOLD–CSF coupling strength in the habit learning system.

Within the habitual learning system (Figure 3B), patients with OCD also exhibited significantly weaker regional BOLD–CSF coupling and negative derivative regional BOLD–CSF coupling strength (*t* = −2.54, *p* = 0.013; *t* = 3.34, *p* = 0.001). Patients with OCD exhibited a significantly longer time lag of regional BOLD–CSF coupling than HCs (*t* = 3.08, *p* = 0.003), whereas no significant between‐group difference was found in the time lag of negative derivative BOLD–CSF coupling (*t* = 1.87, *p* = 0.065).

### 3.4 Associations With Demographic Variables and Clinical Measures

Correlations between gBOLD–CSF coupling strength and age and years of education were examined. Consistent with previous findings, gBOLD–CSF coupling strength tended to weaken with increasing age (*r* = 0.26, *p* = 0.004) (Figure 4A). Significant correlations were also found between global BOLD–CSF coupling strength and years of education (*r* = −0.29, *p* = 0.001) and head motion (*r* = −0.21, *p* = 0.022) (Figure 4B).

**FIGURE 4:**
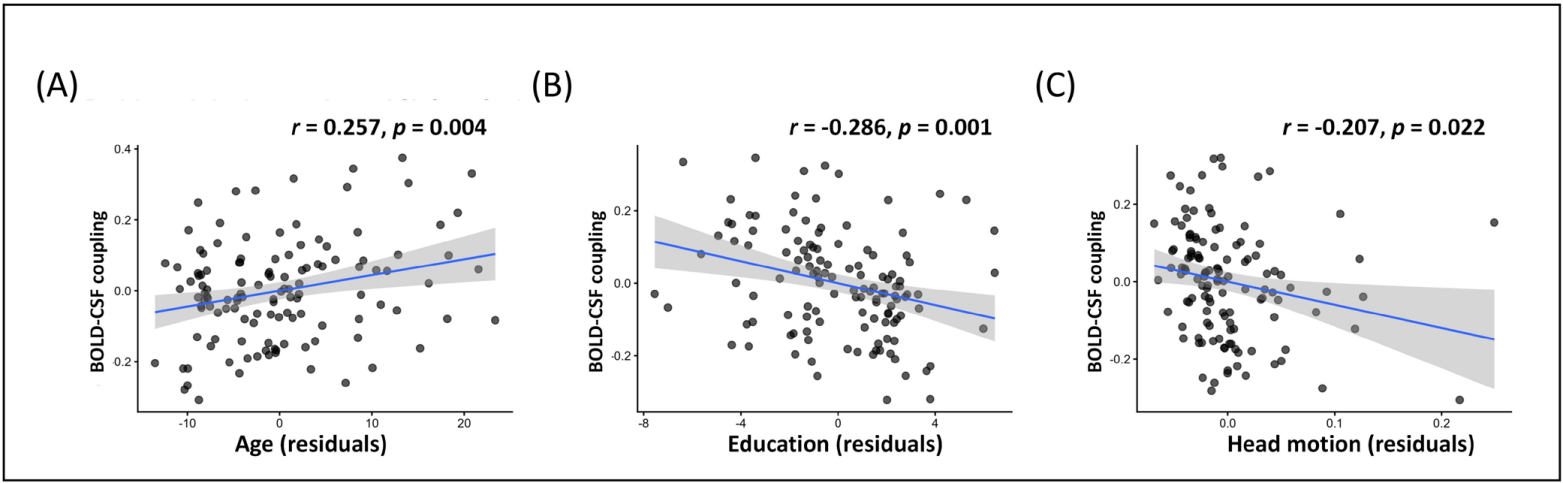
Correlation between demographic characteristics and gBOLD–CSF coupling strength. Scatter plots illustrate partial correlations between demographic variables and regional BOLD–CSF coupling strength after controlling for other covariates. Residualized values were plotted for visualization. (A) Age was significantly negatively correlated with the gBOLD–CSF coupling strength (*r* = 0.26, *p* = 0.004). (B) Years of education were significantly positively correlated with the gBOLD–CSF coupling strength (*r* = −0.29, *p* = 0.001). (C) Head motion was significantly positively correlated with the gBOLD–CSF coupling strength (*r* = −0.21, *p* = 0.022).

Correlations between gBOLD–CSF coupling strength and clinical scale scores were not significant.

## 4 Discussion

In the present study, we investigated CSF dynamics associated with the glymphatic system in patients with obsessive‐compulsive disorder (OCD) by quantifying coupling measures and their corresponding temporal delays at both whole‐brain and system‐specific levels. Compared with healthy controls, patients with OCD exhibited reduced coupling strengths and prolonged temporal lags at both global and regional levels. The weaker BOLD–CSF coupling strength observed in individuals with OCD reflects reduced temporal coordination between hemodynamic oscillations and CSF fluctuations. Furthermore, the results of negative derivative BOLD–CSF coupling focusing on the descending phase of the BOLD signal also support the reduced glymphatic‐related CSF response intensity in patients with OCD. Longer BOLD–CSF coupling time lags in patients with OCD indicate altered temporal responsiveness of CSF dynamics. These findings indirectly suggest glymphatic‐related alterations in patients with OCD.

Previous studies have reported a high prevalence of sleep disturbances in individuals with OCD [50], which may also contribute to glymphatic dysfunction [22]. In addition, chronic stress experienced by patients with OCD may disrupt glymphatic function [51]. Recent studies further elaborated a mechanistic framework linking peripheral infection, central nervous system inflammatory responses, and glymphatic system impairment [25], with reciprocal interactions between inflammation and glymphatic function [52]. Therefore, central nervous system inflammation and peripheral infections in patients with OCD may represent potential factors influencing glymphatic‐related alterations [53]. The environmental exposome framework may provide a broader perspective for understanding glymphatic‐related alterations in OCD. This framework emphasizes that cumulative environmental exposures, including chronic stress, sleep disruption, metabolic factors, and inflammatory stimuli, may interact with biological systems to influence brain imaging phenotypes and psychiatric vulnerability. Therefore, glymphatic‐related CSF dynamics may represent one potential neuroimaging phenotype reflecting the cumulative effects of environmental and biological factors on brain function [54].

The imbalance between the goal‐directed and habitual learning systems is currently a dominant theoretical framework in the study of OCD pathophysiology [55]. Previous studies have suggested that dysfunction within both the goal‐directed and habitual learning systems contributes to symptom persistence [46]. Our regional findings extend this framework by suggesting that alterations in CSF dynamics related to the glymphatic system may be associated with dysfunction within these systems. However, the cross‐sectional nature of this study and its methodological limitations prevent conclusions regarding whether glymphatic‐related alterations contribute to, result from, or co‐develop with abnormalities in these neural systems.

In the present study, we did not observe significant associations between illness duration or age of onset and any of the BOLD–CSF coupling measures. A recent study by Zhang et al. reported that obsessive symptoms in OCD were associated with altered mGluR5 distribution and enrichment of microglia‐related gene signatures [56]. Notably, mGluR5 can form functional macromolecular complexes with aquaporin‐4 (AQP4) and Na^+^/K^+^‐ATPase on astrocytic membranes, which collectively regulate glutamate uptake and glutamate‐induced astrocytic swelling. Meanwhile, microglial activation has been suggested to interact bidirectionally with the glymphatic system [57]. Therefore, glymphatic‐associated abnormalities may contribute to obsessive symptoms through interactions with multiple biological pathways, including inflammatory and immune dysregulation, neurotransmitter imbalance, and disturbances in metabolic homeostasis [10, 58]. However, this proposed mechanism requires further validation using multimodal evidence.

Although not the primary focus of this study, BOLD–CSF coupling strength decreases with increasing age, suggesting an age‐related decline in glymphatic function. Impairment of the glymphatic system may lead to the accumulation of metabolic waste, which has been proposed as an important mechanism through which aging contributes to the development of neurodegenerative diseases [14, 33].

This study has several limitations. First, the lack of inflammatory and immune‐related biomarkers limited our ability to directly characterize the biological mechanisms linking glymphatic dysfunction to OCD pathophysiology. Future studies integrating peripheral and central immune markers are warranted to clarify the potential interplay among chronic stress, immune dysregulation, glymphatic dysfunction, and altered neural dynamics in OCD. Second, sleep quality was not systematically assessed in the present study. Given the proposed role of sleep in regulating glymphatic activity, future studies incorporating objective sleep measurements are needed to determine whether sleep disturbances are associated with altered BOLD–CSF coupling in individuals with OCD. Third, although stringent statistical correction was applied, residual head motion‐related confounds cannot be completely excluded and may influence BOLD–CSF coupling estimates. Future investigations employing advanced denoising approaches and multimodal validation are needed to further establish the robustness and biological specificity of these measures.

## 5 Conclusion

Weaker BOLD–CSF coupling and negative‐derivative BOLD–CSF coupling were observed in patients with OCD compared to HCs, spanning both global measures and regional analyses within the goal‐directed and habitual systems. OCD patients also showed a longer time lag of global BOLD–CSF coupling. These results indicate altered glymphatic‐associated CSF dynamics in OCD, providing novel mechanistic insights into the disorder.

## Author Contributions

Jinqi Liu: data curation, formal analysis, writing – original draft, review and editing. Ziyun Xv: conceptualization, writing – review and editing, methodology. Wanting Liu: writing – review and editing. Carol A. Seger: writing – review and editing. Ziwen Peng: supervision, project administration, funding acquisition, conceptualization, writing – review and editing. Gangqiang Hou: conceptualization.

## Funding

This work was supported by the National Natural Science Foundation of China, 32471141, 32171081. Basic and Applied Basic Research Foundation of Guangdong Province, 2023A1515011782. Striving for the First‐Class, Improving Weak Links and Highlighting Features (SIH) Key Discipline for Psychology in South China Normal University, and the Research Center for Brain Cognition and Human Development, Guangdong, China, No. 2024B0303390003. Medical Scientific Research Foundation of Guangdong Province, A2025094.

## Ethics Statement

The authors affirm that all procedures contributing to this work adhere to the ethical standards of the Institutional Ethics Committee of Shenzhen Kangning Hospital, Shenzhen (Approval No. 2019‐K015‐01), in accordance with the Declaration of Helsinki.

## Consent

Written informed consent was acquired from each participant.

## Conflicts of Interest

The authors declare no conflicts of interest.

## Supporting information


**Figure S1:** Comparison between medicated and unmedicated OCD patients.
**Table S1:** Associations with age of onset and illness duration.
**Figure S2:** Correlation between age of onset and coupling strengths.
**Figure S3:** Individual‐level coupling analysis.
**Table S2:** Group comparisons of demographic characteristics and clinical subscale scores.
**Table S3:** Statistical comparisons of time delays between HC and OCD groups.

## Data Availability

The data that support the findings of this study are available from the corresponding author upon reasonable request.
