# Supplementary material for: Reduced Coupling Between Blood‐Oxygen‐Level‐Dependent Signal and Cerebrospinal Fluid Flow in Obsessive‐Compulsive Disorder
Source: CNS Neurosci Ther. 2026 Nov 9;32(11):e71232. doi: 10.1002/cns.71232 (PMC13748822; doi:10.1002/cns.71232)
Supplement: Supplementary file 1 [file CNS-32-e71232-s001.docx]

1. **Medication Use Analysis**

Given that a substantial proportion of OCD participants in the present study were receiving psychotropic medications, we further examined whether medication status influenced BOLD-CSF coupling measures. Among the medicated patients, 12 received selective serotonin reuptake inhibitors (SSRIs; including sertraline, fluvoxamine, and paroxetine), either as monotherapy or in combination with atypical antipsychotics (e.g., olanzapine, quetiapine, and clozapine). Seven patients received benzodiazepines, and three patients received mood stabilizers. In total, 18 patients were prescribed multiple psychotropic medications. Previous studies have reported that fluvoxamine treatment may affect prefrontal cerebral blood flow in patients with OCD (59) and that SSRIs can influence low-frequency BOLD activity and functional connectivity patterns (60). Therefore, we compared global and regional BOLD-CSF coupling strength between medicated and unmedicated OCD patients.

After controlling for age, sex, education years, and head motion as measure by mean framewise displacement (FD), no significant group differences were observed between medicated and unmedicated OCD patients in any of the three coupling measures (*t_global_* = -0.49, *p* = 0.630; *t_habit-learning_* = -0.22, *p* = 0.828; *t_goal-directed_* = -0.73, *p* = 0.475). These findings suggest that medication status did not significantly affect the observed BOLD-CSF coupling alterations in OCD patients.

**Supplementary Figure 1.** Comparison between medicated and unmedicated OCD patients

2. **Associations with Age of Onset and Illness Duration**

We further investigated whether age of onset and illness duration were associated with glymphatic system-related coupling alterations in OCD patients. Partial correlation analyses were performed between age of onset, illness duration, and the three BOLD-CSF coupling measures while controlling for sex and mean framewise displacement. No significant associations were observed between age of onset or illness duration and any of the three coupling measures.

**Supplementary Table 1.** Associations with age of onset and illness duration

|  | BOLD-CSF coupling strength | *r* | *p* |
| --- | --- | --- | --- |
| Onset | Goal-directed | 0.309 | 0.067 |
|  | Habitual-learning | 0.240 | 0.159 |
|  | Global | 0.204 | 0.232 |
| Duration | Goal-directed | -0.177 | 0.316 |
|  | Habitual-learning | -0.045 | 0.800 |
|  | Global | -0.072 | 0.684 |

**Supplementary Figure 2.** Correlation between age of onset and coupling strengths

3. **Extracted gBOLD Signal by Subject Level**

Following previous studies, the present study primarily used group-averaged cross-correlation curves and negative derivative cross-correlation curves to identify characteristic coupling points (61). Specifically, the peak/valley values of the group-level cross-correlation curves, as well as the cross-correlation values at the +2 s and +4 s time points, were used as the final estimates of BOLD-CSF coupling strength and negative derivative BOLD-CSF coupling strength for all participants. However, this approach does not explicitly account for inter-individual variability in temporal coupling patterns.

Therefore, using global BOLD-CSF coupling as an example, we further extracted subject-specific coupling parameters by identifying the minimum cross-correlation value (valley point) from each participant’s individual cross-correlation curve and negative derivative cross-correlation curve following the method of Yang (62). These individual-level values were used as subject-specific estimates of BOLD-CSF coupling strength and negative derivative BOLD-CSF coupling strength, respectively. We then examined group differences in these subject-level coupling measures between healthy controls and OCD patients.

After controlling for age, sex, years of education, and head motion (mean framewise displacement), a significant group difference was observed in subject-level BOLD-CSF coupling strength (*t* = 2.242, *p* = 0.027), with OCD patients showing significantly reduced coupling strength compared with healthy controls. In contrast, no significant group difference was observed for negative derivative BOLD-CSF coupling strength(*t* = 1.087, *p* = 0.279).

**Supplementary Figure 3.** Individual-level coupling analysis

4. **Group Comparisons of Demographic Characteristics and Clinical Subscale Scores**

As a supplementary presentation of clinical characteristics, we provide detailed group comparisons of clinical measures, including subscale scores. Compared with healthy controls, OCD patients showed no significant difference in the symptom dimension of hoarding. The detailed results are presented in the table below.

**Supplementary Table 2.** Group comparisons of demographic characteristics and clinical subscale scores

|  | **HC（n=74）** | **OCD（n=53）** | ***p*** |
| --- | --- | --- | --- |
| Gender(M/F) | 43/31 | 39/14 | 0.072 |
| Age | 27.22±8.53 | 29.36±8.43 | 0.091 |
| Education years | 13.92±2.37 | 13.02±3.25 | 0.178 |
| Y-BOCS | 5.32±5.62 | 20.22±7.76 | <0.001 |
| Y-BOCS_Obsession | 2.97 ± 3.49 | 9.24 ± 5.35 | <0.001 |
| Y-BOCS_Compulsion | 2.32 ± 3.03 | 7.60 ± 5.28 | <0.001 |
| OCI-R | 12.08±8.11 | 23.64±12.75 | <0.001 |
| OCI-R_Cleaning | 1.75±2.02 | 4.14±3.18 | <0.001 |
| OCI-R_Obsession | 2.12±1.98 | 5.30±2.59 | <0.001 |
| OCI-R_Hoarding | 2.52±1.66 | 2.72±2.73 | 0.635 |
| OCI-R_Ordering | 2.44±1.77 | 3.81±3.15 | 0.029 |
| OCI-R_Checking | 5.32±5.62 | 20.22±7.76 | <0.001 |
| OCI-R_Neutral | 1.91±1.74 | 3.10±2.75 | 0.038 |
| BDI | 6.32±6.29 | 16.04±11.02 | <0.001 |
| S_AI | 36.39±9.47 | 48.35±12.89 | <0.001 |
| T_AI | 38.44±9.34 | 52.08±12.63 | <0.001 |

5. **Selection of** **time lags for extracting coupling strength**

Because some time-lag measures showed between-group differences between HCs and patients with OCD for global and regional BOLD–CSF coupling and negative-derivative gBOLD–CSF coupling, we separately examined the mean time lags in the two groups at each analysis level using a temporal resolution of 2 s. The mean time lags differed by less than 2 s between the two groups at all levels. Therefore, the time lags corresponding to the extrema of the correlations in the two groups were consistently identified at +2 s and −4 s. The extraction method was consistent with Han (63), and the time lags used to quantify the BOLD–CSF and gBOLD–CSF coupling were also in accordance with those of previous studies, making the results comparable across studies (61,64). Therefore, we used a common time lag for both groups when extracting the coupling values (+2 s for BOLD-CSF coupling strength and -4 s for negative-derivative gBOLD-CSF coupling strength) .

The detailed results of between-group comparisons of time lags are presented in the table below.

**Supplementary Table 3.** Statistical comparisons of time delays between HC and OCD groups

|  | Time lag (s)  BOLD-CSF coupling | | | Time lags (s)  negative-derivative BOLD–CSF coupling | | |
| --- | --- | --- | --- | --- | --- | --- |
|  | HC  (M±SD) | OCD  (M±SD) | *p* | HC  （M±SD） | OCD  （M±SD） | *p* |
| Global | 1.51 ± 1.27 | 2.60 ± 2.71 | 0.008 | -3.59 ± 1.84 | -3.32 ± 1.75 | 0.400 |
| Goal-directed | 2.30 ± 2.76 | 2.42 ± 2.70 | 0.811 | -3.24 ± 2.82 | -3.66 ± 1.99 | 0.331 |
| Habit-learning | 1.16 ± 1.59 | 2.53 ± 2.94 | 0.003 | -4.08 ± 1.98 | -3.09 ± 3.45 | 0.07 |

**References**

59. Hoehn-Saric R, Pearlson GD, Harris GJ, Machlin SR, Camargo EE. Effects of fluoxetine on regional cerebral blood flow in obsessive-compulsive patients. Am J Psychiatry. 1991 Sep;148(9):1243–5. doi:10.1176/ajp.148.9.1243 PubMed PMID: 1883007.
60. Wang L, Xia M, Li K, Zeng Y, Su Y, Dai W, et al. The effects of antidepressant treatment on resting-state functional brain networks in patients with major depressive disorder. Human Brain Mapping. 2015 Feb 1;36(2):768–78. doi:10.1002/hbm.22663
61. Han F, Brown GL, Zhu Y, Belkin-Rosen AE, Lewis MM, Du G, et al. Decoupling of global brain activity and cerebrospinal fluid flow in parkinson’s disease cognitive decline. Mov Disord. 2021 Sep;36(9):2066–76. doi:10.1002/mds.28643 PubMed PMID: 33998068; PubMed Central PMCID: PMC8453044.
62. Yang HC (Shawn), Inglis B, Talavage TM, Nair VV, Yao J (Fiona), Fitzgerald B, et al. Coupling between cerebrovascular oscillations and CSF flow fluctuations during wakefulness: An fMRI study. J Cereb Blood Flow Metab. 2022 Jun;42(6):1091–103. doi:10.1177/0271678X221074639
63. Han F, Chen J, Belkin-Rosen A, Gu Y, Luo L, Buxton OM, et al. Reduced coupling between cerebrospinal fluid flow and global brain activity is linked to alzheimer disease–related pathology. PLOS Biology. 2021 Jun 1;19(6):e3001233. doi:10.1371/journal.pbio.3001233
64. Fultz NE, Bonmassar G, Setsompop K, Stickgold RA, Rosen BR, Polimeni JR, et al. Coupled electrophysiological, hemodynamic, and cerebrospinal fluid oscillations in human sleep. Science. 2019 Nov;366(6465):628–31. doi:10.1126/science.aax5440
